# Fast Green FCF Alleviates Pain Hypersensitivity and Down-Regulates the Levels of Spinal P2X4 Expression and Pro-inflammatory Cytokines in a Rodent Inflammatory Pain Model

**DOI:** 10.3389/fphar.2018.00534

**Published:** 2018-05-23

**Authors:** Fang Xu, Jing Yang, Fan Lu, Rongjun Liu, Jinwei Zheng, Junfang Zhang, Wei Cui, Chuang Wang, Wenhua Zhou, Qinwen Wang, Xiaowei Chen, Junping Chen

**Affiliations:** ^1^Ningbo Key Laboratory of Behavioral Neuroscience, Zhejiang Provincial Key Laboratory of Pathophysiology, The Medical School of Ningbo University, Ningbo University, Ningbo, China; ^2^Department of Anesthesiology, Ningbo No. 2 Hospital, Ningbo, China

**Keywords:** fast green FCF, inflammatory pain, inflammation, P2X4, pro-inflammatory cytokines, tumor necrosis factor, interleukin

## Abstract

Fast Green FCF (FGF), a biocompatible dye, recently drew attention as a potential drug to treat amyloid-deposit diseases due to its effects against amyloid fibrillogenesis *in vitro* and a high degree of safety. However, its role in inflammatory pain is unknown. Our study aimed to investigate the effect of FGF in the inflammatory pain model induced by complete Freund’s adjuvant (CFA) and to identify the associated mechanisms. We found that systemic administration of FGF reversed mechanical and thermal pain hypersensitivity evoked by CFA in a dose-dependent manner. FGF treatment decreased purinergic spinal P2X4 expression in the spinal cord of CFA-inflamed mice. FGF also down-regulated spinal and peripheral pro-inflammatory cytokines [tumor necrosis factor-α (TNF-α), interleukin-1β (IL-1β), and interleukin-6 (IL-6)], but did not alter the spinal level of nerve growth factor (NGF) or brain-derived neurotrophic factor (BDNF). In conclusion, our results suggest the potential of FGF for controlling the progress of inflammatory pain.

## Introduction

Chronic inflammation reinforces the pain pathways in the nervous system that can cause the sensation of pain to become exaggerated or inappropriate (Kidd and Urban, 2001). Unfortunately, current treatments for chronic inflammatory pain with non-steroidal anti-inflammatory drugs (NSAIDs) and cyclooxygenase-2 inhibitors (COXIBs) have alarming side effects relating to the gastrointestinal tract, renal, and cardiovascular systems (Moodley, 2008). Consequently, exploring novel and safe treatment modalities is still a considerable need.

Fast Green FCF (FGF) is a triphenylmethane color additive used to color food, drugs, and cosmetics. It is approved by U.S. Food & Drug Administration (FDA) and exhibits a high degree of safety (the acceptable daily intake is up to 25 mg/kg/day in humans) (Borzelleca and Hallagan, 1992). Recently, the role of FGF in protein-aggregation has been investigated. FGF suppresses the generation of amyloid fibrils in lysosomes under acidic conditions, suggesting a potential role of FGF in preventing amyloid-aggregation diseases (How et al., 2016). However, the biological action of FGF and the underlying mechanism in inflammatory pain has not been explored yet.

**FIGURE 1:**
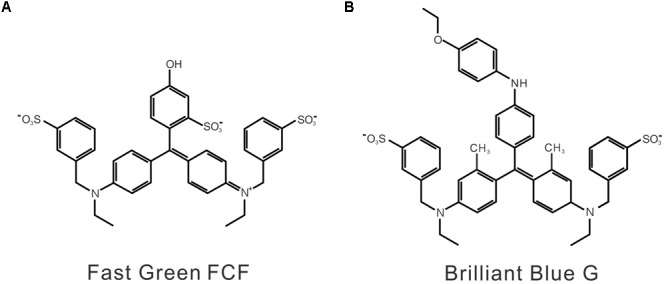
The chemical structures of FGF **(A)** and BBG **(B)**.

Although endogenous targets of FGF in inflammatory pain are unknown, ionotropic purinergic P2X receptors, which are activated by extracellular adenosine, 5-triphosphate (ATP), may be involved in FGF’s action. First, the contribution of P2X receptors to pain sensation and development has been proved by numerous basic and clinical studies (Burnstock, 2016b; Bernier et al., 2017). Among seven P2X receptor subtypes (P2X1–7), P2X4 and P2X7 receptors share a similar protein structure and are the predominant P2X receptor subtypes expressed in immune cells and microglia (Boumechache et al., 2009; Burnstock, 2016b; Bernier et al., 2017). Upregulation of P2X4/P2X7 receptors has been verified in the spinal cord and the peripheral tissue in chronic pain. Genetic deletion or pharmacological blockade of P2X4/P2X7 receptor alleviates inflammatory and neuropathic pain, suggesting the significant contribution of P2X4/P2X7 receptors to chronic pain development (Tsuda et al., 2003; Ulmann et al., 2008, 2010; Trang et al., 2009; Skaper et al., 2010; Trang and Salter, 2012; Jurga et al., 2016, 2017). Second, P2X receptors also play an essential role in amyloid-related pathology (Woods et al., 2016). P2X7 receptor expression is required for microglial activation by amyloid-beta and P2X7 inhibition reduces amyloid plaques in Alzheimer’s disease transgenic mice (Sanz et al., 2009; Diaz-Hernandez et al., 2012; Ni et al., 2013). Third, FGF is a close structural analog to Brilliant Blue G (BBG) (**Figure 1**), which is a potent and non-competitive P2X7 antagonist (Jiang et al., 2000). Similarly, BBG is also a biocompatible dye and could promote the formation of non-toxic amyloid-beta aggregates (Wong et al., 2011). Moreover, the antinociceptive and anti-inflammatory effects of BBG via antagonizing P2X7 receptors have been demonstrated (Skaper et al., 2010; North and Jarvis, 2013). Therefore, we aimed to examine whether FGF alleviates inflammatory pain and modulates P2X receptors.

Extensive evidence highlights a critical role of pro-inflammatory cytokines, such as tumor necrosis factor (TNF)-α, interleukin (IL)-1β, and IL-6, in chronic pain (Clark et al., 2013; Ji et al., 2014). Enhanced release of these inflammatory cytokines has been reported in different types of chronic pain (Backonja et al., 2008; Zin et al., 2010; Lampa et al., 2012). Nevertheless, neurotrophic factors, including nerve growth factor (NGF) and brain-derived neurotrophic factor (BDNF), are also filed as crucial players in chronic pain. Increased spinal NGF and BDNF levels sensitize nociceptors and alter neural plasticity, which in turn exacerbates the painful sensation transmitted to the brain (Nijs et al., 2015; Denk et al., 2017). Notably, the releases of pro-nociceptive cytokines and neurotrophic factors are essential downstream events of P2X4/P2X7 activation during inflammation and chronic pain (Trang and Salter, 2012; Burnstock, 2016a). Thus, we investigated whether the FGF’s action involves regulation of these factors.

In the present study, we explored the effect of FGF on mechanical and thermal hypersensitivity in an inflammatory pain model induced by complete Freund’s adjuvant (CFA). We further examined the involvement of P2X4/P2X7 receptors and the release of pro-inflammatory cytokines and neurotrophic factors to explore the mechanism of its action. Our work contributes to the discovery of potential biocompatible molecules for safe treatment modalities of inflammatory pain.

## Methods

### Animals

Male ICR mice (8–10 weeks, 20–25 g; Experimental Animal Center of Zhejiang Province, China) were used in this study. The animals were housed in a temperature-controlled animal facility with a 12-h light–dark cycle. Water and food were freely available in their home cages. All procedures were approved by the Animal Care and Use Committee of Ningbo University in accordance with the guideline for the Care and Use of Laboratory Animals by National Institutes of Health (NIH Publications No. 80-23).

### Pain Behavioral Tests

The mouse model of inflammatory pain was induced by CFA. Briefly, freshly prepared CFA (20 μl, 50 % in saline) was injected intradermally into the plantar surface of the right hind paw (Cao et al., 1998; Malin and Molliver, 2010; Alhadeff et al., 2018). Pain behavioral tests were conducted before (baseline), 1, 3, 5, 7, and 9 days after the CFA injection. Mice injected with the same volume of saline served as controls. For FGF treatment, freshly dissolved FGF in saline was injected either intraperitoneally daily for 9 days (the first dose of FGF was given immediately after CFA injection) or intradermally into the plantar surface. The CFA and FGF were purchased from Sigma-Aldrich (St. Louis, MO, United States).

#### Measurement of Mechanical Nociception

Mechanical sensitivity was measured on the inflamed hind paw using a series of Von Frey filaments (Stoelting, United States) as previously described (Zhu et al., 2015). Mice were placed in individual transparent Perspex cubicles with a wire mesh bottom and allowed to habituate for at least 20 min. The filaments were applied to the plantar surface of the right hind paw in a series of ascending forces. Each filament was tested five times, and the mechanical threshold was defined as the minimal force that caused at least three withdrawals observed out of five trials.

#### Measurement of Thermal Nociception

A thermal plantar analgesia instrument (Ugo Basile, Italy) was used in this experiment. The infrared red (I.R.) heat intensity of the plantar test instrument was set to 45, and the cut-off latency was set at 25 s to avoid tissue damage in the event of failure to remove the paw. Mice were placed in individual chambers of the glass-floored testing cage for 30 min to acclimate. Paw withdrawal latencies to noxious heat were automatically recorded by applying the I.R. heat stimulus to the inflamed hind paw of each mouse. Each mouse was stimulated three times. The minimal interval between two successive stimuli is 5 min to avoid possible sensitization. The withdrawal latency was defined as the mean value of the three values.

### Enzyme-Linked Immunosorbent Assays (ELISA) Assay

After behavior tests, all animals were anesthetized by CO2 and then decapitated at 9 days after CFA or saline injection. The inflamed tissue of the right hind paw and L4–L6 spinal cord segments were harvested. The samples were rinsed with cold saline and homogenized for ELISA assays of cytokines (TNF-α, IL-1β, and IL-6) and neurotrophic factors (NGF and BDNF). The preparation of all reagents, the working standards, and the protocol was followed according to the manufacturer’s instructions. The absorbance was read at 450 nm using a microplate spectrophotometer (Thermo Inc., United States).

### Real-Time Polymerase Chain Reaction (RT-PCR)

The RT-PCR analysis was performed as previously described (Malin and Molliver, 2010). Briefly, mice were anesthetized with CO_2_ and sacrificed at 9 days after CFA or saline injection. The L4–L6 segments of the spinal cord were collected and frozen in liquid nitrogen. The spinal cord samples were then added to 1 ml Trizol reagent (Invitrogen, Carlsbad, CA, United States) and homogenized. After adding 200 μl chloroform to the homogenate, the mixture was centrifuged at 12,000 *g* for 15 minutes at 4°C. The upper aqueous phase was then transferred to another clean tube and RNA was precipitated with 0.5 ml of isopropanol by centrifuge at 12000 *g* for 15 min at 4°C. The RNA pellet washed with 1 ml 75% ethanol and dissolved in diethyl pyrocarbonate-treated water. RNA purity was determined using the 260-nm absorbance recorded by a spectrophotometer. Two micrograms of total RNA were reverse-transcribed to complementary DNA using Invitrogen Superscript II reverse transcriptase according to the manufacturer’s instructions (Invitrogen, Carlsbad, CA, United States). Negative control reactions were run without mRNA to test for contamination. After PCR amplification, a dissociation curve was plotted against melting temperature to ensure amplification of a single product. Primers for P2X7 was: 5′-AAGTTCCAAGACCCCAGATGGA-3′ (forward), 5′-GCAATTTCCACACTGGCACC-3′ (reverse); for P2X4 was: 5′-GCTGCAGAAAACTTCACCCTC-3′ (forward), 5′-CATGATGCCTCCCTCCACTG-3′ (reverse); for GAPDH (the housekeeper gene): 5′-CATGGCCTTCCGTGTTCCTA-3′ (forward), 5′-TACTTGGCAGGTTTCTCCAGG-3′ (reverse). All primers were designed by Primer-BLAST at the NCBI website as previously described (Ye et al., 2012) and then synthesized by BGI Co. Ltd (Shenzhen, China).

### Western Blot

Mice were anesthetized with CO_2_ and sacrificed at 9 days after CFA or saline injection. The inflamed tissue of the right hind paw and L4–L6 segments of the spinal cord were collected and homogenized in lysis buffer (20 mM Hepes, pH 7.4, 100 mM NaCl, 5 mM EDTA, 1% Triton X-100) containing protease inhibitors (Promega, Madison, United States). Lysates were then centrifuged at 12,000 rpm for 30 min at 4°C. Protein concentration from tissues was determined using BCA Protein Assay Kit (Beyotime, Beijing, China). Samples were separated on 10% SDS-PAGE gels and transferred to PVDF membranes (0.22 μm; Millipore, Temecula, CA, United States). The membrane was blocked with 5% non-fat dry milk and 0.5% Tween 20 in Tris-buffered saline (TBST) at 4°C. The membrane was then incubated overnight at 4°C with rabbit anti-P2X4 (1:100, Alomone Labs, Israel) and mouse anti-β-actin monoclonal (1:2000; Abcam, Cambridge, MA, United States) antibodies. After washes in TBST, the membrane was then incubated with Alexa Fluor 700-conjugated goat anti-rabbit antibody (1:5000; Abcam, Cambridge, MA, United States) for 60 min. Target bands were revealed with a fluorescence scanner (Odyssey Infrared Imaging System, LI-COR Biotechnology, NE, United States). Western blots were analyzed using Image J analysis software (NIH, United States) to quantify the bands.

### Molecular Docking

Molecular docking analyses were accomplished by the SYBYL (Tripos Inc., St. Louis, MO, United States) software and the implanted programs. Three-dimensional crystal structure of ATP-gated P2X4 ion channel was retrieved from the protein data bank (PDB code: 4DW1) (Hattori and Gouaux, 2012). The three-dimension structure of fast green was constructed using standard geometric parameters of SYBYL, and then optimized by Powell method. The Surflex-Dock program, a program used an empirically derived scoring function based on the binding affinities of protein-ligand complexes, was employed to perform docking analysis. As a flexible docking method, Surflex-Dock has been proven to be effective in treating various receptors (Jain, 2003). During the simulations, the rotatable bonds of the ligands were defined whereas the receptor was kept rigid.

### Data and Statistical Analysis

Data are presented as means ± SE. Analyses were performed using the software package GraphPad Prism 5 (GraphPad Software, San Diego, CA, United States). One-way or two-way analysis of variance (ANOVA) was followed by Bonferroni *post hoc* tests as used for analyzing statistic difference as indicated in figure legends. *P* < 0.05 was considered as statistically significant.

## Results

### Systemic Administration of FGF Ameliorated Mechanical Allodynia and Thermal Hyperalgesia Induced by CFA in a Dose-Dependent Manner

**FIGURE 2:**
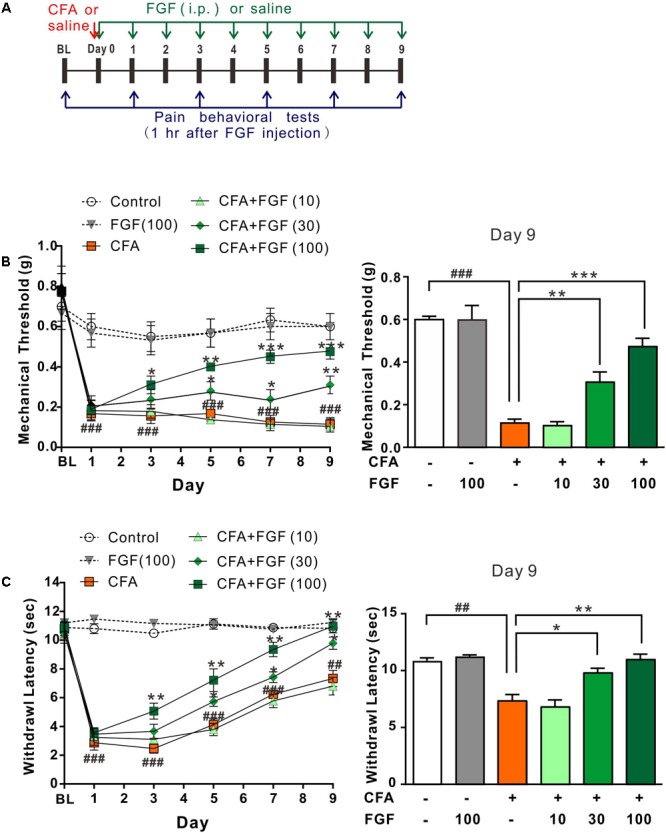
FGF reversed CFA-induced mechanical allodynia and thermal hyperalgesia in a dose-dependent manner. **(A)** The diagram of the experimental timeline. The baseline (BL) threshold was measured at the day before any treatment. On day 0, CFA emulsion or saline was injected into the intradermal region of the left hind paw. Mice then received intraperitoneal (i.p.) FGF or saline injections daily until day 9; the first FGF treatment was applied 1 h after the CFA injection on day 0. Pain behavioral tests were performed 1 hr after FGF treatment. Effects of different doses of FGF (10, 30, or 100 mg/kg) on the mechanical threshold and the withdrawal latency were shown in **(B)** and **(C)**, respectively. Two-way ANOVA and Bonferroni multiple comparison tests as *post hoc* analyses were used (*n* = 7–9 mice per group). ^##, ###^ vs. controls and ^∗, ∗∗, ∗∗∗^ vs. CFA-treated animals. One symbol, *p* < 0.05; two symbols, *p* < 0.01; three symbols, *p* < 0.001.

**Figure 2A** shows the timeline of drug application and pain behavioral experiments. The injection of CFA into plantar tissue causes persistent tactile/mechanical allodynia and thermal hyperalgesia in the injured hind paw (**Figures 2B,C**). Systemic administration of FGF at a dose of 10 mg/kg (daily for 9 days) did not influence the mechanical (**Figure 2B**) and the thermal (**Figure 2C**) hypersensitivity. However, 30 mg/kg or 100 mg/kg FGF alleviated the mechanical (**Figure 2B**) and the thermal (**Figure 2C**) pain since day 5 or day 3 post-CFA injection, respectively. These results exhibit a dose-dependent effect of FGF on CFA-induced pain.

We then examined whether FGF’s action is transient. The pain thresholds were measured one hour before the FGF treatment (23 hours after previous FGF injection). Similarly, 100 mg/kg FGF accumulatively reversed the mechanical allodynia (Supplementary Figure S1B) and thermal hyperalgesia (Supplementary Figure S1C) since day 5 post-CFA injection. The effect of FGF persisted for at least 6 days after the treatment was discontinued (Supplementary Figures S1D,E), suggesting a prolonged and accumulative effect of FGF on inflammatory pain evoked by CFA.

### The Anti-nociceptive Effect of FGF May Not Involve P2X7 Receptor, Although FGF Shares Structure Similarity With P2X7 Antagonist BBG

We next investigated whether FGF’s action involves inhibition of the P2X7 receptor because FGF and BBG share a similar chemical structure. Since high-selective P2X4 agonists are not commercially available (Abdelrahman et al., 2017), we tested whether FGF could block the P2X7-agonist-evoked pain as BBG. Mice first received an intradermal injection of a selective P2X7 agonist 2′(3′)-O-(4-Benzoylbenzoyl)-ATP (Bz-ATP) and then injections of FGF, BBG, or A804598 (a high-selective P2X7 antagonist) (Supplementary Figure S2A). A single injection of Bz-ATP induced mechanical and thermal hyperalgesia for at least 7 days (Supplementary Figures S2B,C). However, neither an intradermal injection nor repeated intraperitoneal injections of 100 mg/kg FGF reduced the Bz-ATP-evoked mechanical (Supplementary Figure S2B) or the thermal hyperalgesia (Supplementary Figure S2C). In contrast, either BBG or A804598 abolished Bz-ATP-evoked pain (Supplementary Figures S2D,E). The results indicate that FGF has an analgesic mechanism different from BBG, although they are close structural analogs and both ameliorate CFA-evoked inflammatory pain.

### FGF Treatment Down-Regulated P2X4 mRNA Transcription and Protein Expression in the Spinal Cord of CFA-Inflamed Mice

**FIGURE 3:**
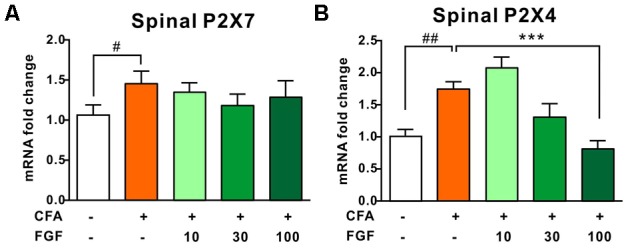
FGF down-regulated the spinal P2X4 mRNA level post-CFA injection, but did not affect P2X7 mRNA transcription. The spinal P2X7 **(A)** and P2X4 **(B)** mRNA levels in the L4–L6 spinal segments were analyzed by real-time PCR. All data are normalized to controls. One-way ANOVA and Bonferroni multiple comparison tests as *post hoc* analyses were used (*n* = 7 per group). ^#, ##^ vs. controls and ^∗, ∗∗, ∗∗∗^ vs. CFA-treated animals. One symbol, *p* < 0.05; two symbols, *p* < 0.01; three symbols, *p* < 0.001.

Several lines of evidence have verified the upregulation of P2X4 and P2X7 transcription in response to inflammation (Bernier et al., 2017). In accordance with previous investigations, CFA-evoked inflammation elevated the mRNA levels of both receptors in the lumbar spinal cord (**Figures 3A,B**) (Burnstock, 2016a; Bernier et al., 2017). FGF treatment did not influence the P2X7 mRNA level (**Figure 3A**), but down-regulated the P2X4 mRNA level in a dose-dependent manner (**Figure 3B**), indicating an association between FGF and P2X4 transcription.

**FIGURE 4:**
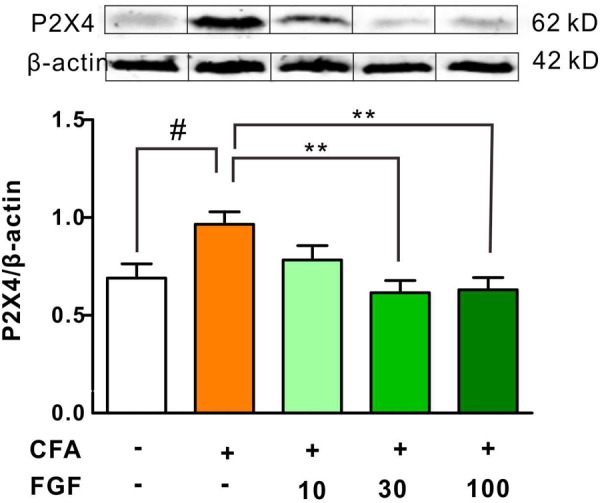
FGF decreased the P2X4 protein expression in the spinal cord of CFA-inflamed mice. The protein levels of P2X4 receptors in the lumbar spinal cord were evaluated by western blot. The top panel: representative western blot images indicating the P2X4 and β-actin protein bands. The bottom panel: quantification of the fold change of P2X4/β-actin. One-way ANOVA and Bonferroni multiple comparison tests as *post hoc* analyses were used (*n* = 5–6 per group). ^#, ##^ vs. controls and ^∗, ∗∗, ∗∗∗^ vs. CFA-treated animals. One symbol, *p* < 0.05; two symbols *p* < 0.01; three symbols *p* < 0.001.

It has been documented that nerve injury upregulates spinal P2X4 expression (Tsuda et al., 2003; Ulmann et al., 2008, 2010). Thus, we further examined the P2X4 protein expression in response to inflammation. We found that CFA treatment enhanced spinal P2X4 expression (**Figure 4**). However, CFA did not alter P2X4 protein expression in the inflamed paw tissue or lumbar dorsal root ganglia (data not shown), indicating that CFA-induced inflammation mainly affects P2X4 expression in the spinal cord but not the peripheral tissue. Consistent with mRNA and behavioral data, FGF at the dose of 30 or 100 mg/kg diminished both spinal P2X4 expression on day 9 after CFA injections, but 10 mg/kg FGF did not affect spinal P2X4 expression (**Figure 4**).

### FGF Down-Regulated the Spinal and the Peripheral Levels of Pro-inflammatory Cytokines Post-CFA Injection

**FIGURE 5:**
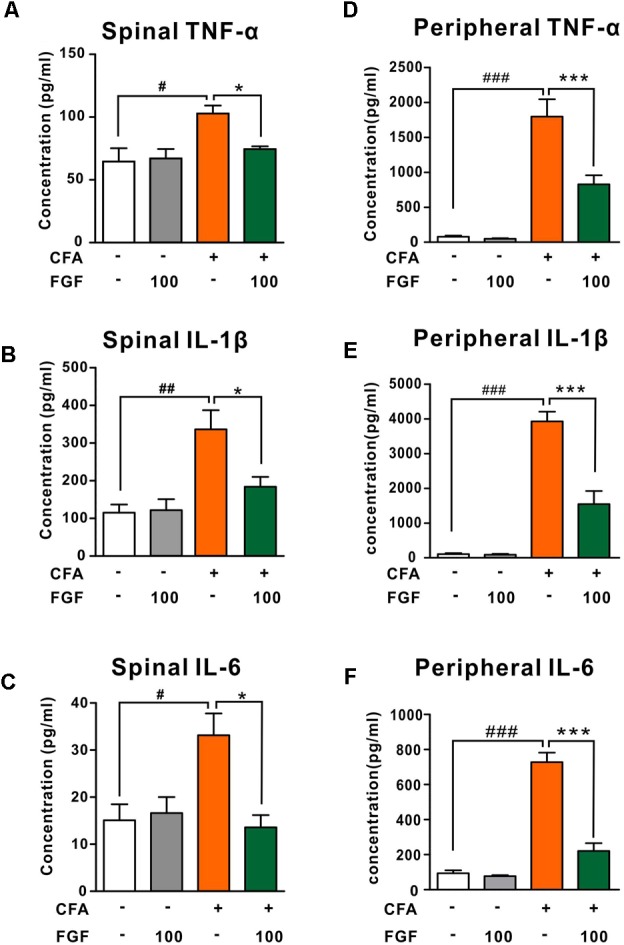
FGF down-regulated TNF-α, IL-1β and IL-6 levels in the lumbar spinal cord in the spinal cord and the paw tissue of CFA-inflamed mice. The levels of TNF-α, IL-1β, and IL-6 in the lumbar spinal cord **(A–C)** and the inflamed paw tissue **(D–F)** were examined by ELISA. ^#, ##, ###^ vs. controls and ^∗, ∗∗, ∗∗∗^ vs. CFA-treated animals. One symbol, *p* < 0.05; two symbols, *p* < 0.01. One-way ANOVA and Bonferroni multiple comparison tests as *post hoc* analyses were used (*n* = 7–9 mice per group).

The contribution of pro-inflammatory cytokines to chronic pain development has been confirmed. It is also reported that activation of P2X4 receptors triggers the release of pro-inflammatory cytokines in the spinal microglia after nerve injury (Jurga et al., 2017). We thus examined the effect of FGF on the release of TNF-α, IL-1β, or IL-6 in the spinal cord and the paw tissue. FGF treatment lowered levels of TNF-α, IL-1β, and IL-6 both in the spinal cord (**Figures 5A–C**) and the paw tissue of CFA mice (**Figures 5D–F**), suggesting an anti-inflammatory activity of FGF in CFA-induced inflammation.

### FGF Did Not Affect the NGF or BDNF Level in Lumbar Spinal Cord Post-CFA Injection

**FIGURE 6:**
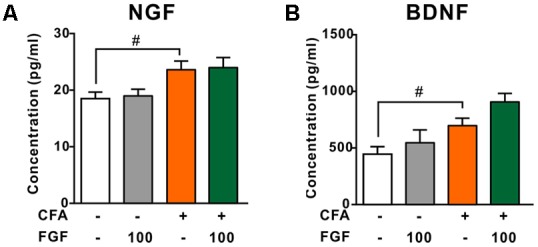
The effect of FGF on spinal NGF and BDNF levels post-CFA injection. The levels of NGF **(A)** or BDNF **(B)** in the lumbar spinal cord were examined by ELISA. ^#^ vs. controls and ^∗^ vs. CFA-treated animals. One symbol, *p* < 0.05; one-way ANOVA and Bonferroni multiple comparison tests as *post hoc* analyses were used (*n* = 7–9 mice per group).

Growing evidence suggests that elevated NGF and BDNF levels are hallmarks of chronic inflammatory pain (Taves et al., 2013; Denk et al., 2017). NGF triggers the release of spinal cytokines in chronic inflammation (Denk et al., 2017). In addition, activation of P2X4 receptors leads to the release of BDNF from microglia after peripheral nerve injury (Ulmann et al., 2008; Trang et al., 2009). Hence, we examined the effect of FGF on the spinal NGF and BDNF levels. However, neither the NGF (**Figure 6A**) nor the BDNF (**Figure 6B**) level in the lumbar spinal cord was affected by FGF, indicating that the anti-nociceptive and anti-inflammatory effects of FGF may not associate with the production of spinal NGF and BDNF.

### The Molecular Docking Simulation Indicates an Interaction Between P2X4 Ion Channel and FGF

**FIGURE 7:**
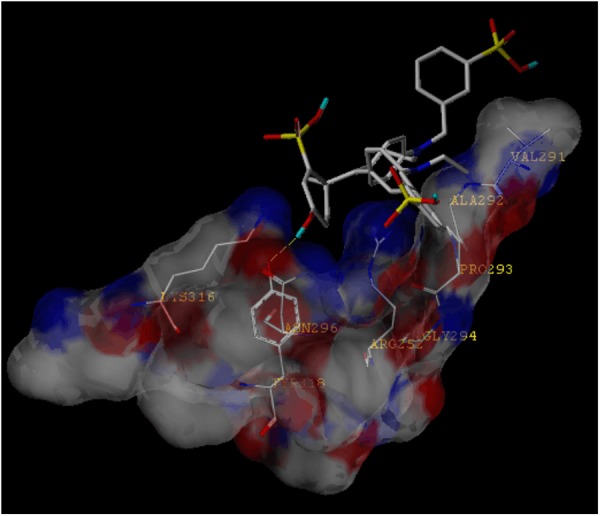
Molecular docking simulation of the interaction between FGF and the P2X4 receptor. The low-energy conformation of fast green bound to P2X4 ion channel generated by molecular docking. FGF is depicted as a stick model showing carbon (white), oxygen (red), nitrogen (dark blue), sulfate (yellow), and hydrogen (light blue). Yellow dash lines represent hydrogen bonds.

Because the structure of FGF is similar to BBG, and the structure of P2X4 is similar to P2X7, FGF may bind the P2X4 receptor. We thus applied a molecular docking simulation to predict any potential interaction between FGF and the P2X4 receptor. Molecular docking analysis of the low-energy conformation of FGF predicts that FGF may form hydrogen bonds with side chains of Asn296 in P2X4 ion channel (**Figure 7**). The simulation result suggests that FGF may be a low-to-moderate affinity modulator for the P2X4 receptor. We cannot exclude the possibility that FGF may modulate the P2X4 receptor indirectly.

## Discussion

In the present study, we evaluated the effect of FGF on CFA-induced inflammatory pain. FGF significantly alleviated CFA-induced pain hypersensitivity. FGF also down-regulated the expression of the P2X4 receptor, as well as the production of pro-inflammatory cytokines, in the spinal cord and the peripheral inflamed tissue. We found that the anti-nociceptive effect of FGF was accumulative and prolonged. Repeated FGF administration (100 mg/kg; ≥3 days) was sufficient to alleviate CFA-induced pain, and the effect persisted after the discontinuation of daily treatment (Supplementary Figure S1). A possible interpretation is that FGF needs to accumulate in the system to exert its anti-nociceptive effect, such as by reducing the central inflammatory response or altering the neural plasticity of spinal nociceptors. Consequently, our study shows that multiple administration of FGF (>30 mg/kg) can gradually ameliorate chronic inflammatory pain, suggesting that FGF might serve as an anti-inflammatory supplement to help control inflammatory pain or accelerate the healing of chronic inflammation.

Growing evidence suggests a crucial role of the P2X4 receptor in chronic pain and inflammation. The P2X4 receptor is mainly expressed in microglia and immune cells (Stokes et al., 2017). In neuropathic pain, P2X4 receptors are expressed *de novo* by activated microglia in the spinal cord after peripheral nerve injury (Ulmann et al., 2008), and P2X4 knockout mice display impaired spinal inflammasome activation after spinal cord injury (de Rivero Vaccari et al., 2012). In inflammatory pain, P2X4 knockout mice exhibit attenuations of tactile allodynia and also of the CFA-induced swelling of the hind paw (Tsuda et al., 2009). These findings are in agreement with our results showing enhancement of spinal P2X4 expression in CFA-inflamed mice. Since knockdown of P2X4 receptors has been proved to be sufficient to suppress inflammatory and neuropathic pain (Tsuda et al., 2009; Jurga et al., 2016, 2017), the anti-nociceptive effect of FGF may be achieved by modulating P2X4 expression. Although our docking simulation suggests that FGF is a low-to-moderate affinity P2X4 modulator and may not directly block the P2X4 receptor, there is evidence that P2X4 receptor function is related to its transcription and expression levels. Previous investigations demonstrated that P2X4 expression at the membrane is limited and the majority of P2X4 receptors are localized in endosomes and lysosomes in resting cells (Robinson and Murrell-Lagnado, 2013). In lipopolysaccharide (LPS)-induced inflammation, current responses of P2X4 receptors in BV-2 murine microglial cells are enhanced, and the facilitation of P2X4 function is concomitant with higher P2X4 transcription and protein expression (Raouf et al., 2007). Therefore, it is possible that FGF facilitates internalization and degradation of P2X4 receptors and subsequently reduces inflammatory pain

The downstream signal pathways of P2X4 during inflammatory pain still need to be clarified. Ulmann et al. (2010) have shown that P2X4 knockout mice exhibit a complete absence of inflammatory prostaglandin E2 (PGE_2_) in tissue exudates. Stimulating P2X4 receptors triggers calcium influx, p38 MAPK phosphorylation and cytosolic phospholipases A2 activation, indicating a role of the P2X4-p38MAPK-PGE2 pathway in peripheral inflammation (Ulmann et al., 2010). The effect of FGF may thus relate with inhibition of PGE_2_ pathway in chronic inflammation. On the other hand, the contribution of the P2X4-BDNF cascade to neuropathic pain has been reported. NGF and BDNF are essential pain mediators that sensitize nociceptive terminals, induce sprouting of nociceptors, and elevate the expression of multiple nociceptive receptors and transmitters (Smith, 2014; Nijs et al., 2015; Denk et al., 2017). Activation of the P2X4 receptor, which is necessary for neuropathic pain development, results in a rapid release of BDNF from microglia after nerve injury (Ulmann et al., 2008; Trang et al., 2009). However, we found that FGF did not reduce the NGF and BDNF levels in the spinal cord. Our findings suggest that the P2X4-BDNF cascade is not involved in the anti-nociceptive effect of FGF on CFA-evoked pain. The discrepancy could be due to different pain models (inflammatory pain vs. neuropathic pain). In inflammatory pain, activation of P2X4 receptors may not promote the release of spinal BDNF. It is also possible that FGF modulates other BDNF-independent downstream pathways in the spinal cord.

The prolonged and accumulative effect of FGF on inflammatory pain indicates that the FGF’s effect may associate with neuroplasticity. It has been reported that P2X4 receptors are involved in neuroplasticity in the CNS. Phosphorylation of the NMDA receptor subunit NR1 in spinal dorsal horn neurons was impaired in P2X4 knockout mice with neuropathic pain (Ulmann et al., 2008). P2X4 knockout mice also display abnormal subunit composition of glutamate ionotropic receptors and impaired dopamine homeostasis in the central nervous system (Wyatt et al., 2013; Khoja et al., 2016). Although the mechanism of FGF’s action is not well understood, the evidence provides possible connections among FGF, P2X4, and neuroplasticity because glutamate and dopamine are essential neurotransmitters modulating neuroplasticity in physiological and pathological states. Additionally, we found that CFA-induced inflammatory pain mainly increased P2X4 expression in the spinal cord but not in the peripheral tissue, indicating differential responses of spinal and peripheral P2X4 receptor to inflammatory pain. The influence of FGF on the spinal P2X4 expression support the hypothesis that FGF affects pain-related neuroplasticity at the spinal level.

Mounting evidence reveals that pro-inflammatory cytokines influence sensory transduction and synaptic plasticity of nociceptors and recruit immune cells. Genetic knockdown or neutralizing these cytokines has been demonstrated to be analgesic (Ren and Torres, 2009; Hess et al., 2011; Taves et al., 2013). Therefore, down-regulation of peripheral and spinal pro-inflammatory cytokines (TNF-α, IL-1β, and IL-6) by FGF could be a possible mechanism of its anti-nociceptive action. In addition, the spinal P2X4 receptor is a crucial player in pain-related inflammatory responses. P2X4 knockout mice show a decreased level of spinal IL-β and the reduced infiltration of spinal neutrophils and monocyte-derived M1 macrophages after spinal cord injury (de Rivero Vaccari et al., 2012). Blockade of P2X4 receptors prevents MMP-9 activation and pro-nociceptive interleukin (IL-1β, IL-18, IL-6) releases in the dorsal spinal cord in the CCI model (Jurga et al., 2017), suggesting that the anti-inflammatory effect of FGF on the spinal cytokines may involve downregulation of spinal P2X4 receptors.

In conclusion, our results demonstrate that FGF alleviates chronic inflammatory pain. The mechanism of its action may involve inhibition of P2X4 expression and downregulation of inflammatory cytokine levels in the spinal cord. Our results suggest that FGF has the potential for the treatment modalities of inflammation and pain associated with chronic inflammation.

## Author Contributions

FX contributed to animal experiments, analysis and interpretation of the data, and drafted the manuscript. JY, FL, and RL contributed to animal experiments, analysis and interpretation of the data, and the writing of the manuscript. JiZ, JuZ, CW, WZ, and QW contributed to molecular experiments, analysis and interpretation of the data, and the writing of the manuscript. WC contributed to the molecular docking experiment, analysis and interpretation of the data, and the writing of the manuscript. XC supervised the study and contributed to the conception and design of the study, the analysis and interpretation of the data and the writing of the manuscript. JC supervised the study and contributed to the conception and design of the study. All authors approved the final version of the manuscript.

## Conflict of Interest Statement

The authors declare that the research was conducted in the absence of any commercial or financial relationships that could be construed as a potential conflict of interest.
