# Supplementary material for: Fast Green FCF Alleviates Pain Hypersensitivity and Down-Regulates the Levels of Spinal P2X4 Expression and Pro-inflammatory Cytokines in a Rodent Inflammatory Pain Model
Source: Front Pharmacol. 2018 May 23;9:534. doi: 10.3389/fphar.2018.00534 (PMC5974208; doi:10.3389/fphar.2018.00534)
Supplement: Supplementary file 1 [file Data_Sheet_1.DOCX]

Supplementary Material

Fast green FCF alleviates pain hypersensitivity and down-regulates the levels of spinal P2X4 expression and pro-inflammatory cytokines in a rodent inflammatory pain model

Fang Xu^1^, Jing Yang^1^, Fan Lu ^1^, Rongjun Liu^1^, Jinwei Zheng^2^, Junfang Zhang^1^, Wei Cui^1^, Chuang Wang^1^, Wenhua Zhou^1^, Qinwen Wang^1^, Xiaowei Chen^1^*, Junping Chen^2^*

^1^Ningbo Key Laboratory of Behavioral Neuroscience, Zhejiang Provincial Key Laboratory of Pathophysiology, School of Medicine, Ningbo University, Ningbo, China

^2^ Department of Anesthesiology, Ningbo No. 2 Hospital, Ningbo, China

*** Correspondence:**Xiaowei Chen
[chenxiaowei@nbu.edu.cn](mailto:chenxiaowei@nbu.edu.cn)

Junping Chen
[13858222873@163.com](mailto:13858222873@163.com)

**Supplementary Figure 1.** The prolonged effect of FGF on CFA-induced pain sensitivity.

The experimental timeline is shown in (A). Pain behavioral tests were performed 1 hr before FGF treatment (100 mg/kg). FGF treatment gradually increased the mechanical threshold (B) and the withdrawal latency (C) in CFA mice. FGF treatment for 9 days (the grey boxes) had prolonged effect on the mechanical threshold (D) and the withdrawal latency (E) in CFA mice until the 15th day. Data are presented as means ± SE. ##, ### vs controls and *, **, *** vs CFA-treated animals. One symbol p < 0.05, two symbols p < 0.01, three symbols p < 0.001; Two-way ANOVA and Bonferroni multiple comparison tests as post hoc analyses were used (n= 7-9 mice per group,).

**Supplementary Figure 2.** The effect of FGF, BBG and A804598 on the Bz-ATP-evoked pain.

A) The diagram of the experimental timeline. The baseline (BL) pain threshold was measured at the day before any drug treatment. Bz-ATP (0.1 mM, 20 μl) or saline was injected into the intradermal region of the left hind paw on day 0. Pain behavioral tests were performed 1 hr after FGF/BBG treatment. The left panel: Mice received either an intradermal (i.d.) injection of FGF (2.5mM, 20 μl) immediately after Bz-ATP or i.p. injections of FGF (100 mg/kg) daily until day 7; the first FGF injection was applied immediately after Bz-ATP at day 0. The right panel: Mice received either an i.d. injection of A804598 (2.5mM, 20 μl) or i.p. injections of BBG (100 mg/kg) daily till day 3; the first BBG or A804598 injection was applied as FGF. FGF did not affect Bz-ATP-evoked mechanical allodynia (B) and thermal hyperalgesia (C). BBG or A804598 completely blocked Bz-ATP-evoked mechanical allodynia (D) and thermal hyperalgesia (E). Two-way ANOVA and Bonferroni multiple comparison tests as post hoc analyses were used (n= 6-7 per group). Data are presented as means ± SE. *, **, *** vs Bz-ATP treated animals. One symbol p < 0.05, two symbols p < 0.01, three symbols p < 0.001.
